# A Clinical and Psychopathological Approach to Radicalization Among Adolescents

**DOI:** 10.3389/fpsyt.2022.788154

**Published:** 2022-04-25

**Authors:** Nicolas Campelo, Alice Oppetit, Caroline Thompson, David Cohen, Estelle Louet

**Affiliations:** ^1^Service de Psychiatrie de l'Enfant et de l'Adolescent, Hôpital Pitié-Salpêtrière, APHP.SU, Paris, France; ^2^Laboratoire PCPP- EA 4056, Institut de Psychologie, Université de Paris, Paris, France; ^3^Institut des Systèmes Intelligents et de Robotiques, Sorbonne Université, Paris, France

**Keywords:** adolescence, radicalization, psychiatric disorder, psychopathology, ideology, violence, family history, trauma

## Abstract

Recent studies have shown higher rates of radicalization of adolescents than in the 2000s. Since 2015, radicalization prevention units have been implemented in child and adolescent psychiatry departments in France. We aimed to report on the psychopathology of adolescents who were followed up in a university department due to their “radical conduct.” Based on the available clinical data (from child psychiatry consultations, long-term family and/or individual therapy, and psychological testing) for 20 adolescents with “radical conduct,” we examined the nature of their radical conduct, their psychopathology, their family characteristics, and the existence or absence of traumatic experiences. Among the 20 adolescents, 4 had radical conduct associated with a delusional syndrome (schizophrenia or a psychotic episode after substance abuse). For the other 16, we found no psychotic conditions. The analysis of other data showed that the adolescents shared some characteristics, such as an important prevalence of intrafamilial violence, sexual abuse, imprisonment of family members, traumatic family histories, and significant psychological control or dependence phenomena occurring in divided families. This diversity of psychopathologies appears consistent with previous studies highlighting the relevance of diverse profiles depending on the presence of a delusional syndrome, the individual's gender and the individual's attraction to violence. Finally, we discuss some psychopathological hypotheses and make therapeutic recommendations. We believe that child and adolescent psychotherapy/psychiatry has a role to play in countering violent extremism by offering adolescents a way out of radical commitment.

## Introduction

Terrorism and radicalization have mostly been studied by researchers in political science, sociology, and criminology within the past two decades (1–3). This research has been conducted mostly in response to security, political, and judicial needs. The field of social psychology has also made significant advances by studying group dynamics and the potential benefits for an individual who joins a violent extremist group (4, 5). Over the last decade, the number of psychology and psychiatry studies has increased; these studies have tried to portray the psychological characteristics of individuals engaged in violent extremism (6–9). Two principal issues have emerged from the findings: 1) the links between mental health and radicalization and 2) the links between psychological characteristics and the trajectories of radicalized individuals.

There is a consensus among the majority of academics regarding the links between mental health and radicalization (10–12). The variability of what is qualified as “mental illness” and “radicalization” in different studies has resulted in reports of heterogeneous prevalence rates of radicalization among individuals with mental illness, ranging from 0 to 57% (11). By closely examining the available data in the literature, Gill et al. estimated that the most accurate rate of radicalization for individuals with a confirmed diagnosis is 14.4%.

Several studies have made some distinctions, resulting in the elaboration of categorizations or profiles. First, Merari distinguished between “suicide bombers” and “organizers” of suicide attacks (13). By comparing organizers and candidates for a suicide attack, he showed that the latter had a significantly lower level of self-esteem, displaying more symptoms of depression and a more dependent and avoidant personality. This psychological profile made individuals more amenable to group, leader, and public influence. Several studies have also shed light on mental health issues being more common for lone actor terrorists (7, 14). Lone actor terrorists were found to exhibit more mental health problems than terrorists who acted in a group and to have relationships with a violent extremist group member but to not truly belong to the group.

However, the associated factors and trajectories of individuals involved in radicalization and terrorism in Western societies depend on the local context (15). Over the last decade, many young people in Europe have subscribed to the ideology of the Islamic State. They have shown their adherence in different ways: travelling to take part in the Iraqi-Syrian armed conflict, spreading the movement's underlying ideas, and participating in violent attacks (16). Unlike Al-Qaeda's terrorism in the 2000s, this radicalization has been an endogenous phenomenon in that young individuals have turned against the environment in which they grew up (17, 18). Data indicate three new characteristics in the radicalization phenomenon in Europe: compared to subjects who joined Al-Qaeda in the 2000s, the recent radicalized subjects include more adolescents, more women, and more recent converts to Islam (3, 10, 19–23). Several scientists have explained these observations based on societal changes, organizational shifts in jihadist organizations, and the growing presence and recruitment activity of such organizations on the internet (18, 24).

These recent findings on youth radicalization have resulted in new methods of prevention. These methods include secondary prevention (directed at individuals identified as being at risk of radicalization) and tertiary prevention (directed at individuals who have planned or committed a violent extremist act). Educational, psychological, and psychiatric programmes have published results highlighting the specificities of these methods (6, 20, 25–27). A longitudinal study found that a worse status at follow-up (still being radicalized or having reached the Islamic State) was associated with neighborhood/proximal phenomena (having attempted to radicalize other relatives and having a close friend or relative imprisoned before radicalization) (25). A comparison of minors vs. adults demonstrated that minors presented more self-harm history before radicalization, fewer attempts to radicalize their surroundings and peers, fewer cases of radicalization among their surroundings and peers, and less radicalization through meeting in person (27). Looking deeper at motivational dimensions, researchers also noticed that some female subjects had a specific affective motivation (looking for an idealistic, religious man to marry). Additionally, male subjects reporting violent tendencies, interest in weapons, adventure, fighting (“male values”), lack of self-esteem, or a lack of interest in searching for affection experienced significantly worse outcomes (20, 25). Another study that focused on imprisoned adults interested in violent extremism established 4 profiles (6): “ambitious offenders,” “criminal network converts,” “people in precarious situations,” and “people who are severely mentally ill.” Individuals in the “severely mentally ill” profile represented 10.7% of the sample and had a mean age of 34 years, which was noticeably older than the mean age of the sample in the previously mentioned study (mean age = 23.32 years). Overall, based on these studies, we hypothesized two main trajectories among European adolescents and young adults involved in radicalization: 1) individuals who radicalized themselves in a neighborhood/proximal context and were drawn to violence and fighting and 2) younger individuals who presented with more psychological vulnerabilities (self-harm history). In this second trajectory, we noticed a better response to deradicalization programmes and, for females, an affective motivational dimension. Nevertheless, mental illness *per se* does not seem to be present in adolescents and young adults, while it appears to be more frequent in adults.

From a more clinical and psychopathological angle, psychiatrists and psychologists who have cared for adolescents and young adults have tried to explain adherence to violent ideologies. Adolescence has been underlined as a period when youth are vulnerable to radicalization (28–30). The importance of family dynamics has also been mentioned (31–33). One of the main hypotheses is that radicalized adolescents or young adults experience psychological control by their parents within the family group and, paradoxically, develop a similar relationship with the radical group: they undo the family's psychological control to recreate it with a group that considers them adults (31, 33).

The main difficulty that we face as care professionals is that quantitative and qualitative studies do not mesh well together (34). On the one hand, global results provide partial indications about mental illness in specific samples, and on the other hand, case studies describe detailed personal trajectories but lack information about representativeness. This conundrum makes it difficult to determine the primary characteristics of the individuals following these hypothetically different trajectories. Is the radicalization process different in an adolescent affected by mental illness than in an adolescent not affected by mental illness? Is it different when the adolescent is a girl rather than a boy? Are there specific trajectories of radicalization for more worrisome and violent adolescents? Based on a psychopathological approach, is it possible to understand the way adolescence and the radicalization process interact with each other? Is it possible to identify signs of psychological control both in adolescents' families and extrafamilial relationships?

The current work is based on 6 years of experience with a radicalization prevention programme in adolescent psychiatry. We aimed to verify the consistency between the characteristics of individuals we provided care for and the literature's main hypotheses on adolescent radicalization (e.g., specific trajectories for adolescents with mental illness, for girls and for worrisome and violent individuals; role played by the adolescence period in the radicalization process; relationships marked by psychological control). In this way, we expected to test these hypotheses on our sample and extend the literature based on clinical insights. By doing so, we hoped to improve the dialogue between international research and caregivers in the field. To meet that objective, we conducted a retrospective study on relevant medical and psychological records and used two complementary evaluation methods: 1) we explored charts with a grid we developed based on the characteristics found in the literature about radicalization among adolescents, and 2) we performed a psychopathological analysis based on a psychodynamic approach.

## Methodology

In this section, we present the setting of this retrospective study. First, we describe the radicalization context among French adolescents. Second, we describe the consultations with the adolescents included in this study. Then, we present the inclusion criteria, sample selection and ethics. Finally, we introduce i) the retrospective grid we built and used to identify characteristics of interest (radical conduct, psychiatric assessment, family group characteristics and traumatic experiences) and ii) the psychopathological analysis based on a psychodynamic perspective (speech in relation to radical conduct, ideology and group; relational modalities, conflict management, and defense mechanisms; and perception of ideals and symbolic laws).

### The Radicalization Context Among French Adolescents

In France, a radicalization prevention policy was launched in 2015 (35). Establishing the exact number of French adolescents who have adopted a violent ideology is difficult. Referring to a Senate report from August 2016, 364 minors were reported by judicial authorities (21). Unfortunately, we lack details regarding this report's criteria. In addition, prosecution linked to violent ideologies is mostly for “advocating for terrorism” (AT) and “criminal association to commit terrorism” (AMT or “*Association de Malfaiteur en vue d'une entreprise Terroriste*” in French). A recent study of the Judicial Youth Protection Service (PJJ, or *Protection Judiciaire de la Jeunesse* in French) indicated that the PJJ oversaw 80 adolescents who were prosecuted for AMT between 2012 and 2020. This means that in this period of 8 years, 80 adolescents adopted a violent ideology and had contact with members of a terrorist organization. This suggests that violent radicalization among adolescents remains marginal if we consider the French population rate of 4.161.115 in 2021 for young people aged 15–19 (36).

### Consultations for Radicalization in the Child Psychiatry Department

Since November 2015, the child psychiatry department of La Pitié-Salpêtrière Hospital in Paris has received adolescents and their families who may be concerned about radicalization. As we explain below, the different definitions of “radicalization” mostly have a sociological background, which makes this concept complicated to work with from a clinical perspective. Additionally, the medicalization and the political use of the term “radicalization” in the last decade has made the term emotionally charged, leading to collective representations of what is supposed to be a “radicalized” individual. Our experience working with these adolescents helped us to dispel our prejudices and to note that a wide range of situations can be called or designated as “radicalization” by parents, relatives, schoolteachers, etc. We chose to provide care for all individuals referred to us with psychological suffering related to “radicalization,” even if their situations did not match the different definitions of this term. Additionally, we explained to the families that our objective was not to address security concerns, as we were careers. However, if there were serious concerns about a violent act, the families could always contact security services if not already done so. To prevent any stigmatization effect, we cared for these adolescents and their families in our child psychiatry department like any other patients without any “radicalization” concerns. The care they received was protected under medical confidentiality.

Youths were referred to these consultations through four key channels: their own parents; the children's welfare service; the police department; and the PJJ, which follows adolescents for AMT or AT. Occasionally, other childhood psychiatric services ask for our help in support of their care. We followed 69 patients from November 2015 to May 2021. The primary care they received depended on their needs and could include a psychiatric assessment, a psychological assessment, family therapy, individual therapy, and/or hospitalization. We identified four different situations in the consultation process: 1) subjected radicalization (children brought to the Iraqi-Syrian war zone by their parents); 2) delusional radicalization (radical ideas/behaviors in the context of a delusional psychotic syndrome); 3) possible radicalization (adolescents who converted to Islam and came from non-Muslim families); and 4) recognized radicalization (adolescents who identified with a violent ideology and were in contact with individuals belonging to a terrorist organization) (37).

### Inclusion Criteria

The most common definition of “radicalization” in France is the definition developed by the sociologist Khosrokhavar: “*A process by which an individual or group adopts a violent form of action, directly linked to an extremist ideology with a political, social or religious content that undermines the established political, social or cultural order*” (18). This definition has limits regarding the scope of prevention. Indeed, most “radicalized” individuals encountered during prevention campaigns have never committed violent acts and therefore do not match this definition. Each situation is different, and the possibility of an actual realization of this act is variable and difficult to identify. These very different scenarios all fall under the same unequivocal and vague “radicalized” designation (37).

The diversity of these situations led us to distinguish individuals who raised concerns about their “radicalization” among those around them from individuals joining an ideology that encourages the use of violence. We adopted the concept of “*radical conduct”* to define the latter group of individuals: “*conduct breaking with past conduct and transgressing a family prohibition, as well as societal laws, that relies on an ideological construction that encourages the use of violence*.” On this basis, we excluded cases in which the adolescent solely converted to a religion different from their families and displayed no evidence of encouraging the use of violence, as well as cases in which parents imposed the violent ideology.

### Sample Selection

We retrospectively selected all adolescents who exhibited “radical conduct” as previously described. We decided to focus on interventions that had to be ended or interrupted in May 2021. We did so to distance ourselves from the ongoing follow-ups and to collect more material.

**Figure 1:**
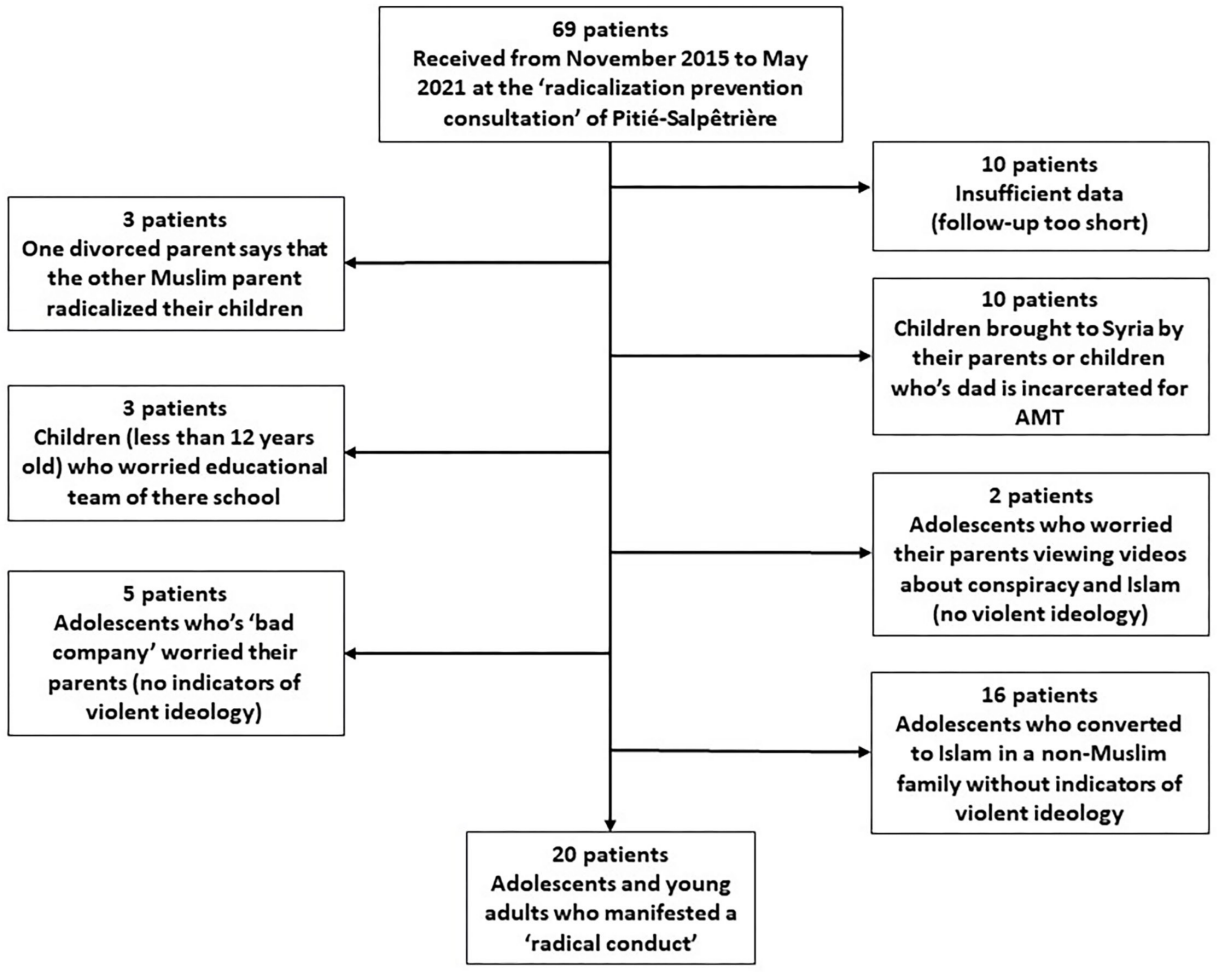
Flow diagram of the study.

The flow diagram is portrayed in Figure 1. From November 2015 to May 2021, we completed clinical care with 69 adolescents suspected of having been involved in radicalized activities. The most common reasons for exclusion were adolescents from non-Muslim families having converted to Islam and not manifesting violent behavior or encouraging the use of violence (*N* = 16); children being brought to Syria by their parents or having fathers who were incarcerated for AMT (*N* = 10); and insufficient data (e.g., the follow-up period was too brief) (*N* = 10). Ultimately, 20 adolescents fulfilled our inclusion criteria and exhibited radical conduct. They ranged in age from 14 to 20 years when they showed radical conduct. The sample was composed of 4 females and 16 males.

### Ethics

Guided by the hospital's Clinical Research Department (DRC), we followed the specific ethical steps of a retrospective study based on the patients' medical records. The Ethics Evaluation Committee of the INSERM (*Institut National de la Santé et de la Recherche Médicale*) Institutional Review Board (IRB00003888, IORG0003254, FWA00005831) reviewed and approved the research project. We also completed a Privacy Impact Assessment validated by the Data Protection Officer of the Public Hospitals of Paris, leading to the registration of the study (n°20190820183539). Finally, we sent an information letter to patients, and to their parents when they were under 18 years old, providing details about the study and giving them the opportunity to contact us if they wanted to object to the use of their medical information.

### Development of the Retrospective Grid

All 20 subjects had child psychiatric consultations and follow-ups. Nicolas Campelo (a psychologist) and Alice Oppetit (a senior psychiatrist) built an analysis grid following several publications offering a clinical and/or descriptive approach of adolescents meeting our inclusion criteria. To construct the grid, first, Campelo and Oppetit selected several characteristics of interest identified in the literature and put them into a table. When a table with these characteristics had been established, we noted whether these characteristics were present for each subject we included (see Tables 1–4). The grid included a list of characteristics identified regarding sociodemographic traits and education (20, 25, 26, 38) and qualitative psychopathology (29–32, 39–42). Campelo and Oppetit homogenized the characteristics identified by each author. Then, they removed the characteristics that they could not evaluate retrospectively with available data. After developing a first analysis grid, they started to complete the grid for each individual. Missing data were too numerous regarding some specific characteristics (e.g., lack of a detailed systemic analysis of family dynamics or adolescents' sexual interests and activities, if any), so these characteristics were discarded. Sociodemographic, conduct, and clinical characteristics were organized into the following four themes: i) radical conduct, ii) psychiatric assessment, iii) family group characteristics, and iv) traumatic experiences. David Cohen (a senior psychiatrist and university professor) and Estelle Louët (a psychologist and university senior lecturer) checked each analysis grid to validate the sociodemographic and clinical data. For the evaluation of family group characteristics, we mostly relied on psychiatric consultations and follow-ups from adolescents and their families. We also used the discussion notes we had compiled with the multidisciplinary teams involved in each treatment (the educational and psychological teams from the PJJ or the children's welfare service or other psychiatric teams caring for the adolescents). Regarding the assessment of the presence or absence of psychological control in the family group, we based our evaluations on the definition given by Barber (43). Then, Nicolas Campelo and Caroline Thompson (a psychologist and expert in family therapy) separately evaluated the presence of psychological control for each adolescent. The other members of the research team checked the only case on which Campelo and Thompson disagreed to reach a consensus. Finally, for the “traumatic experience” part of the study, we used the criteria highlighted by the Adverse Childhood Experiences (ACEs) Scale (44).

**Table 1:** Radical conduct.

**Case**	**Gender**	**Age**	**Radical behaviors**	**Police charge**	**Online contact**	**Physical encounter**	**Converted to Islam**
1	F	14	Had virtual exchanges with an ISIS recruiter and physical meetings with other individuals sharing her ideology; engaged in radical proselytizing on the internet; recruited peers into the group; made plans to go to the Iraqi-Syrian war zone; announced plans on the internet to carry out attacks.	AMT	Yes	Yes	Yes
2	F	14	Had virtual exchanges with an ISIS recruiter; engaged in radical proselytizing on the internet; recruited peers into the group; made plans to go to the Iraqi-Syrian war zone; announced plans on the internet to carry out attacks.	AMT	Yes	No	Yes
3	F	16	Had a physical encounter with an ISIS recruiter and physical encounters with others who share her ideology; engaged in radical proselytizing on the internet; recruited peers into the group; planned attacks registered on the internet; attempted to go to the Iraqi-Syrian war zone.	AMT	Yes	Yes	No
4	F	16	Had internet exchanges with an ISIS recruiter imprisoned in France; identified with a radical ideology; verbalized plans to go to the Iraqi-Syrian war zone.	AMT	Yes	No	No
5	M	14	Made bomb threat calls to train stations and called the French Department of Homeland Security threatening to cut off the head of the Prime Minister in the name of ISIS and threatening to rape the woman telephone operator.	AT	No	No	No
6	M	14	Had virtual exchanges with an ISIS recruiter and physical encounters with others who shared his ideology; recruited peers into the group; engaged in radical proselytizing on the internet; made plans for attacks reported on the internet.	AMT	Yes	Yes	Yes
7	M	14	Had virtual exchanges with an ISIS recruiter; planned attacks registered on the internet; initiated a violent act with a knife but stopped himself.	AMT	Yes	No	No
8	M	15	While hospitalized in a child psychiatry unit, said anti-Semitic things; supported the 9/11 attacks and said he wanted to take up arms to defend Muslims and kill Jews.	No	No	No	Yes
9	M	15	Had virtual exchanges with an ISIS recruiter; planned attacks registered on the internet; initiated a violent act with a knife but stopped himself.	AMT	Yes	No	No
10	M	15	Had virtual exchanges with an ISIS recruiter; engaged in radical proselytizing on the internet.	AMT	Yes	No	No
11	M	15	Went with his two older brothers to the Iraqi-Syrian war zone, and they returned on their own a year and a half later.	AMT	Yes	Yes	No
12	M	15	Before the radical behaviour, organized a false bomb alarm at a train station. Then, had virtual exchanges with an ISIS recruiter; engaged in radical proselytizing on the internet; planned attacks registered on the internet; had physical encounters with others who shared his ideology; was arrested during an attempt to go to the Iraqi-Syrian war zone; and started a violent act with a knife but was interrupted by the police.	AMT	Yes	Yes	No
13	M	16	While hospitalized in adult psychiatry, was very hostile toward the medical staff and called them miscreants and racists and threatened them several times with repercussions in the name of Islam.	No	No	No	No
14	M	16	Had virtual exchanges with an ISIS recruiter; engaged in radical proselytizing on the internet; verbalized a plan to go to the Iraqi-Syrian war zone.	AMT	Yes	No	No
15	M	16	Had virtual exchanges with an ISIS recruiter; engaged in radical proselytizing on the internet; verbalized a plan to go to the Iraqi-Syrian war zone; announced plans on the internet to attack the homosexual community.	AMT	Yes	No	No
16	M	16	Had virtual exchanges with an ISIS recruiter; engaged in radical proselytizing on the internet; verbalized a plan to go to the Iraqi-Syrian war zone.	AMT	Yes	No	No
17	M	17	Had virtual exchanges with an ISIS recruiter and physical encounters with others who shared his ideology; engaged in radical proselytizing on the internet; verbalized a plan to go to the Iraqi-Syrian war zone.	AMT	Yes	Yes	Yes
18	M	18	Watched audio-visual content promoting violence in the name of Islam on the internet; engaged in radical proselytizing in his environment; attempted suicide via defenestration while screaming ‘Allahu Akbar'.	No	No	No	Yes
19	M	19	Was introduced to the ideology by his sister; had virtual exchanges with and made money transfers to members of ISIS in Syria.	AMT	Yes	No	Yes
20	M	19	He watched content promoting violence in the name of Islam on the internet and told his mother he wanted to take up arms against the ‘racist French'.	No	No	No	Yes

*AMT, criminal association to commit terrorism; AT, advocating for terrorism*.

**Table 2:** Psychiatric assessment.

**Case**	**Psychiatric diagnosis**	**Social isolation before radical behaviour**	**Conduct disorder (other than radical conduct)**	**Drug abuse**	**Risky sexual behaviour**	**Self-harming behaviour**	**Eating disorder**
1	No psychotic condition	Yes	Yes (transgression)	No	No	No	Yes (bulimia)
2	No psychotic condition	Yes	Yes (transgression)	No	No	No	No
3	No psychotic condition	Yes	Yes (transgression)	No	Yes	No	No
4	No psychotic condition	Yes (also bullying at school)	No	No	Yes	Yes (scarification, suicide attempt)	No
5	No psychotic condition	Yes (also bullying at school)	Yes (transgression, aggression and robbery)	No	No	No	No
6	No psychotic condition	Yes (also bullying at school)	Yes (transgression and aggression)	Yes	Yes	No	No
7	No psychotic condition	No	Yes (transgression, aggression and robbery)	Yes	Yes	Yes (recurring suicidal thoughts)	No
8	Delusional syndrome, schizophrenia	Yes	No	No	No	No	No
9	No psychotic condition	Yes	Yes (transgression)	Yes	No	No	No
10	No psychotic condition	Yes	No	No	No	No	Yes (bulimia)
11	No psychotic condition	No	No	No	No	No	No
12	No psychotic condition	Yes	Yes (transgression and aggression)	Yes	Yes	Yes (suicide attempt)	Yes (bulimia)
13	Delusional syndrome at due to substance	Yes	Yes (transgression and aggression)	Yes	No	No	No
14	No psychotic condition	No	Yes (transgression and aggression)	No	No	No	No
15	No psychotic condition	Yes (also bullying at school)	No	No	No	No	No
16	No psychotic condition	No	Yes (transgression, aggression and robbery)	Yes	No	No	No
17	No psychotic condition	Yes	Yes (transgression and aggression)	No	No	No	No
18	Delusional syndrome, schizophrenia	No	No	No	No	Yes (suicide attempt during delusional syndrome)	Yes (bulimia)
19	No psychotic condition	No	No	No	No	No	No
20	Delusional syndrome, schizophrenia	No	No	No	No	No	No

**Table 3:** Family characteristics.

**Case**	**Family structure**	**Cultural background**	**Do the parents' have health issues?**	**Family drug/alcohol abuse**	**Family members' imprisonment**	**Traumatic death of a relative**	**Domestic violence (committed by...)**	**Family division (division setting)**	**Extremely conflictual family environment**	**Intrafamilial psychological control**
1	Parents are married	Single (France)	No	Yes	Yes	Yes	Yes (father)	Yes (nuclear family vs. the rest of the family)	Yes (generalized intrafamilial and extrafamilial conflict)	Yes
2	Parents are married	Single (North Africa)	No	No	No	No	0	Yes (nuclear family vs. the rest of the family)	No	Yes
3	Parents are divorced	Single (North Africa)	No	No	No	No	Yes (father)	Yes (mother's family vs. father's family)	Yes (between parents due to divorce)	Yes
4	Parents are married	Single (North Africa)	Yes	No	No	Yes	No	No	No	No
5	Parents are divorced	Single (North Africa)	No	No	No	No	No	Yes (mother's family vs. father's family)	Yes (generalized intrafamilial and extrafamilial conflict)	Yes
6	Parents are divorced	Single (France)	No	No	No	No	No	Yes (mother's family vs. father's family)	Yes (between parents due to divorce)	Yes
7	Parents are married	Single (North Africa)	No	Yes	Yes	Yes	Yes (father and brother)	No	Yes (between the family and a person or institution)	Yes
8	Single mother (father died)	Single (East Asia)	Yes	No	No	Yes	No	Yes (mother and child vs. the rest of the family)	No	No
9	Parents are divorced	Mixed (Overseas France and sub-Saharan Africa)	No	Yes	Yes	No	No	Yes (nuclear family vs. the rest of the family)	Yes (between the family and a person or institution)	Yes
10	Parents are married	Single (North Africa)	No	No	No	No	No	No	No	Yes
11	Parents are divorced	Single (North Africa)	No	No	No	No	No	Yes (mother's family vs. father's family)	Yes (between parents due to divorce)	Yes
12	Parents are divorced	Single (North Africa)	No	No	No	Yes	Yes (father)	Yes (mother's family vs. father's family)	Yes (between parents due to divorce)	Yes
13	Single mother (father died)	Single (North Africa)	No	Yes	Yes	Yes	No	Yes (mother's family vs. father's family)	Yes (between parents due to divorce)	No
14	Divorced parents	Single (North Africa)	No	No	Yes	Yes	Yes (Father)	Yes (mother's family vs. father's family)	Yes (between parents due to divorce)	Yes
15	Divorced parents	Single (North Africa)	Yes	Yes	No	No	No	Yes (mother's family vs. father's family)	Yes (between parents due to divorce)	No
16	Single mother (absent father)	Mixed (France and sub-Saharan Africa)	Yes	No	No	No	No	Yes (mother and child vs. the rest of the family)	Yes (between the family and a person or institution)	Yes
17	Single mother (absent father)	Single (France)	No	Yes	No	Yes	Yes (grandfather)	Yes (mother and child vs. the rest of the family)	No	Yes
18	Married parents	Single (France)	No	No	No	Yes	No	No	No	Yes
19	Married parents	Single (France)	No	No	No	No	No	Yes (sister's new family vs. the rest of the family)	Yes (between sister and parents)	Yes
20	Single mother (absent father)	Single (Overseas France)	No	No	No	No	No	Yes (mother's family vs. father's family)	No	No

**Table 4:** Traumatic experiences.

**Case**	**Type I trauma (traumatic death of a relative, having been assaulted and/or viewing very violent videos as a child)**	**Type II trauma** **(domestic violence, extremely conflicting family setting, school bullying, emotional deprivation, repeated sexual abuse, extreme family poverty, father's imprisonment, father's drug abuse, family abandonment and/or mother's psychiatric condition)**	**Sexual abuse (only extrafamilial)**	**Painful grieving**
1	No	Yes	Suspected	No
2	No	Yes	No	No
3	No	Yes	No	No
4	Yes	Yes	No	No
5	No	Yes	Suspected	No
6	No	Yes	Yes	No
7	Yes	Yes	Yes	Yes
8	No	Yes	No	Yes
9	No	Yes	No	No
10	No	No	No	No
11	No	No	No	No
12	No	Yes	Yes	No
13	No	Yes	No	No
14	Yes	Yes	No	Yes
15	No	Yes	No	No
16	No	Yes	No	No
17	Yes	Yes	No	Yes
18	Yes	No	No	Yes
19	No	Yes	No	No
20	Yes	Yes	No	No

### Psychopathological Analysis

As in previous clinical studies (45), we based our psychopathological analysis on a psychodynamic approach, adopting a processual clinical research method to analyses the underlying challenges of symptomatic expressions of radical conduct. We sought to avoid a sterile opposition between clinical and research methods (46). This psychopathological perspective allows for a transnosographic analysis, overcoming the divide between the approach of positing conduct to be completely detached from any psychiatric illness and the approach of integrating conduct into a broader category of depressive- or anxiety-related conditions, as appears in the literature (13, 47). The psychodynamic approach aims to open up to reflection not centered on categories but rather on the processes involved in the contemporary expression of suffering in adolescence. Such an approach allows for the consideration of the specificities of adolescence as the subject constructs his or her identity, undergoing multiple changes in the process. To effectively understand behavior, it is important to consider it within the context of a continuum between normal and pathological behavior rather than a dichotomy between normal and pathological, which would tend to exaggerate the pathological side. From our view, arguing for a continuum between normal and pathological behavior and deploying a psychodynamic approach privileges a processual interpretation of the symptoms, thus overcoming the stasis of an approach based only on nosography.

We based the psychopathological analysis on the clinical and psychopathological literature (28, 29, 31, 39) and our previous clinical observations (22, 33, 37, 48). After identifying the main themes of interest, we chose to merge them based on the following logic. First, we decided to analyses the adolescents' speech about their radical conduct, the ideology they had chosen and the group with which they shared this ideology. We focused on the way these adolescents related to their symptoms because it gave us information about the reasons why they had engaged in radical conduct and what they felt about these radical behaviors. Second, we decided to analyses the relational modalities, conflict management and defense mechanisms these adolescents manifested during care because this radical conduct was in opposition to the environment in which they grew up. We also bore in mind that the literature has linked radicalization with the adolescent period as a difficult period of life constituted by desires and aggressiveness. The purpose of this second theme was to describe the way these adolescents related to their environments (relatives, friends, professionals), the way they psychologically managed conflicts and the way they managed the desires and frustrations they felt in relation to others (defense mechanisms). The last theme was about the way the adolescents related to ideal and symbolic laws. We chose this theme because these adolescents had adopted an ideology that removed most of the limitations of French society by promising a grandiose purpose and by imposing horrible demands in the name of this ideal. This led us to interrogate the way these adolescents relate to ideals and whether they had correctly integrated symbolic laws.

In summary, we established the following three main themes: i) speech in relation to radical conduct, ideology and group; ii) relational modalities, conflict management, and defense mechanisms; and iii) perception of ideals and symbolic laws. We based this analysis on psychiatric interviews as well as on long-term family and/or individual therapy. We also based our analysis on psychological tests (49): the Weschler scale (an IQ test designed to measure intelligence and cognitive ability) (50) and the Rorschach test and Thematic Apperception Test (projective tests using psychological interpretation that examine a person's personality and emotional functioning) (51). We used these tests as part of a psychopathological analysis, described in the second part of the results section. We used a qualitative method based on phenomenological analysis to report this section based on the work of Campelo, Louët and Thompson by reading the adolescents' charts and testing summaries (52). Of note, Louët has expertise in psychodynamic theory (53), and Thompson has expertise in family therapy (54).

## Results

### Characteristics Identified With the Retrospective Grid

In this part, we present the results we obtained using the retrospective grid. The characteristics are organized into the four following themes: i) radical conduct, ii) psychiatric assessment, iii) family group characteristics, and iv) traumatic experiences.

#### Radical Conduct

**Figure 2:**
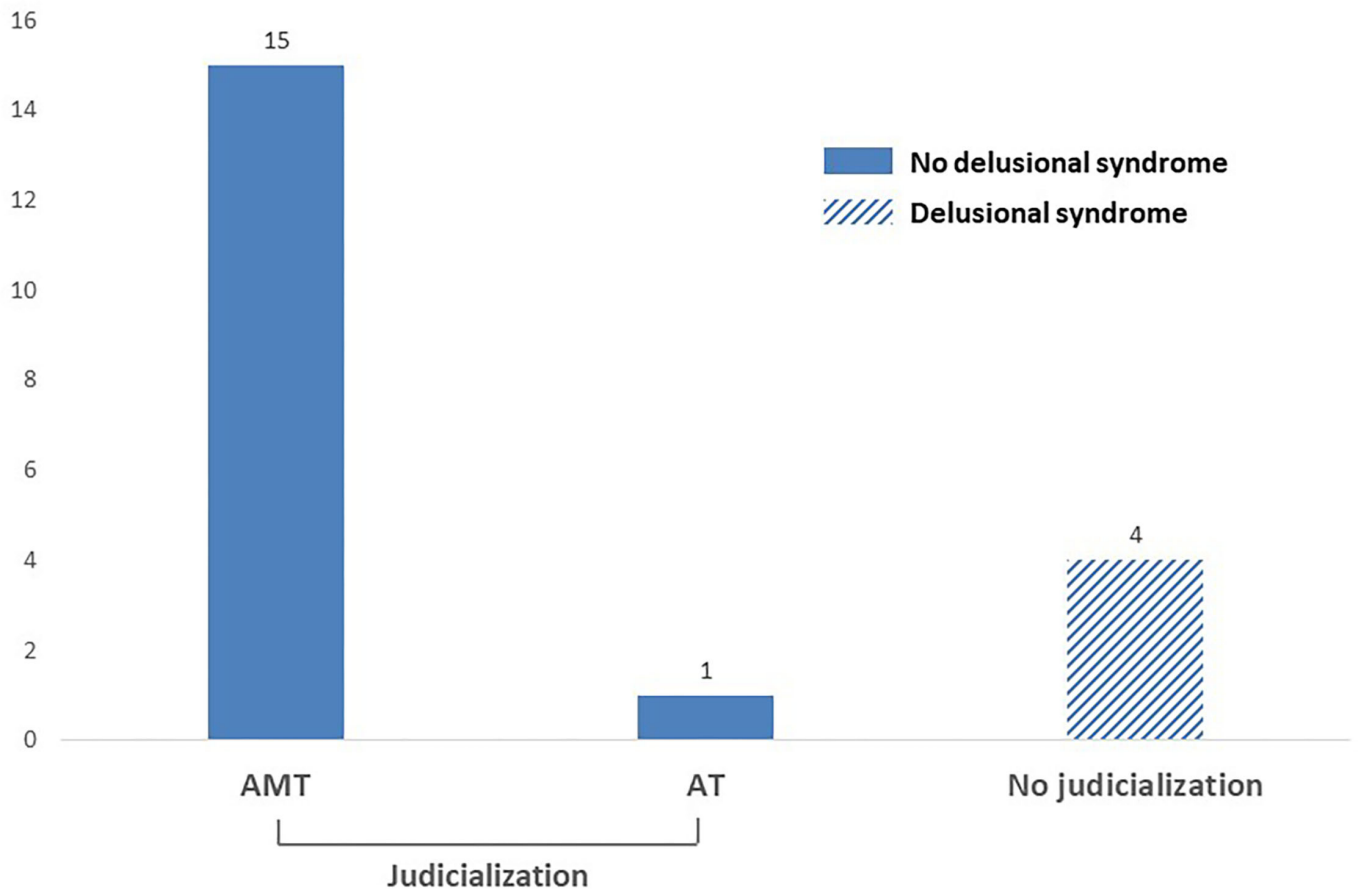
Radical conduct with and without delusional syndrome.

As shown in Table 1, radical conduct was apparent in our sample, and a first examination led us to distinguish two groups. We had four subjects whose radical conduct began while they exhibited a delusional syndrome (Figure 2). They claimed the use of violence in the name of Islam but were not prosecuted for their behavior by the Justice Department. The other group consisted of 16 adolescents convicted for their radical conduct. For 15 of them, the police and the justice department identified a direct link with individuals encouraging the use of violence in the name of their ideology. Thus, they were convicted for AMT. Finally, one adolescent (Subject 5, Table 1) was j convicted for AT.

All of the subjects had accessed Islamic content on the internet; 15 (all convicted for AMT) had online contact with at least one member of a terrorist organization, but only 6 of them had physical contact. Additionally, two female adolescents introduced several friends to the violent ideology by integrating them into the group. Ten youths had clearly expressed an intention to go to Syria, and four had tried to leave: three were stopped, and one (Subject 11) reached Syria but came back to France by himself, running away from war. Seven of these 15 subjects had exchanged communications on the internet concerning a proposed or planned attack. Three of them had initiated a violent act with a knife, two of them had stopped themselves before choosing a victim on the street, and another one was arrested by the police before committing an act he had planned to carry out as part of a group. Two adolescents had prescribed to the violent ideology without planning to leave the country or commit violent acts.

Regarding religious beliefs, eight youths from the whole sample had decided to convert to Islam, while the others had grown up in a Muslim family. Interestingly, a majority of individuals from the “delusional” group had converted to Islam (3 out of 4), whereas less than a third of the convicted individuals had done so (5 out of 16).

#### Psychiatric Assessment

Regarding the four youths presenting with delusional syndrome, three had schizophrenia, and one had a psychotic condition post-drug exposure. All four presented social isolation due to their psychiatric conditions, and one adolescent (Subject 18) expressed suicidal intentions. Subject 13 had different characteristics, as he suffered from serious social issues linked to a personal history of family estrangement. He had multiple addictions and expressed radical opinions after a delusional syndrome caused by his use of drugs. We diagnosed a conduct disorder characterized by temporary violent behaviors, as well as several incidents of wandering and running away starting with the onset of adolescence. For all four subjects, the resolution of the delusional syndrome co-occurred with the disappearance of radical speech. We noticed that the subjects' radical conduct, mostly characterized by radical proselytism, appeared with the typical clinical picture of a delusional syndrome. The intensity of their radical proselytism was related to the intensity of their delusional syndrome, and both reduced gradually after they received antipsychotics. The rate of this decline obviously depended on the psychiatric disorder they had and the specific medication they received, but we noticed that the antipsychotics clearly reduced radical proselytism that was related to delusional beliefs and the certainty the subjects had about them (Table 2).

Other adolescents displayed a combination of various psychiatric conditions and problematic behaviors. Eleven presented with a conduct disorder, 10 had social anxiety disorder with social isolation before the appearance of their radical conduct, 5 had suffered from drug abuse, 5 had engaged in risky sexual behaviors, 3 had suicidal tendencies (ideas and/or attempts), and 3 had eating disorders (specifically bulimia). By analyzing the 11 adolescents with conduct disorders, we found that 4 youths showed a transgression against parental rules (e.g., running away); 4 had committed other aggressions; and 3 had combined transgression, aggression, and robbery. Physical aggressiveness toward others only appeared in male subjects. Female subjects exhibited bravado, destructive behaviors, and often a form of escapism. Drug use and abuse mostly co-occurred for subjects with conduct disorders characterized by aggressiveness toward others. Risky sexual behaviors occurred for subjects who had experienced (or were suspected of having experienced) sexual abuse (Subjects 3, 4, 6, 7, 12). In addition, the three subjects who expressed suicidal intentions had also engaged in risky sexual behaviors (Subjects 4, 7, 12). Suicidal intentions emerged in different forms (Subject 4 showed scarification and a suicide attempt, Subject 7 showed a fascination by death and suicidal ideas, and Subject 12: had attempted suicide but denied it).

#### Family Characteristics

For the adolescents who had displayed radical conduct during a delusional syndrome, their family structures showed the following characteristics. Three had an absent father, including two whose fathers had died before they were 2 years old. They had diverse cultural origins, but both parents came from the same cultural background. Additionally, we identified a psychiatric pathology for one youth's mother, as well as drug addiction and imprisonment for the deceased father of another adolescent. As far as we know, there was no domestic violence, either for this adolescent or in his family history (Table 3).

Concerning the 16 adolescents who were convicted for their radical conduct, only 6 had parents who still lived together. Most of them (eight subjects) had parents who were separated. Two subjects lived only with their mother because of their father's early abandonment. Fourteen families had the same cultural background (4 from France and 10 from North Africa), and only 2 of them had multiple origins (France, countries in sub-Saharan Africa, and Overseas France).

Several subjects had traumatic family histories, including health issues (*N* = 2), psychiatric conditions (one subject's mother had psychiatric pathology, and six subjects had family members with drug abuse), membership in a religious cult (*N* = 1, Subject 2), a very painful or traumatic death of a relative (*N* = 6), family members' imprisonment (*N* = 4), and domestic violence (*N* = 6). These last two last variables need to be compared with known French statistics. The level of imprisonment was limited to 0.1% in the overall French population in 2021 (55). Similarly, the estimated frequency of domestic physical or sexual violence was 2.2% in 2010 (56).

The analysis of the family dynamics showed evidence of intrafamilial disruption for 13 families. For these families, relations between members were interrupted (complete absence of contact) or extremely hostile. In addition, 12 of the 16 subjects had grown up in an extremely conflictual family environment, which often creates serious loyalty conflicts for the adolescent. In addition, we observed frequent intrafamilial psychological control experienced early on by the youths (*N* = 14), who suffered from psychological control, manipulation, and/or excessive pressure from a member of their family circle, and they were not able to defend themselves or ask for help. Intrafamilial violence or psychological pressure in the context of parents' separation could be humiliating. In many cases, we observed an excessively present parent having a “symbiotic relationship” with the adolescent and pushing him or her to adopt specific and expected points of view or behaviors.

In sum, all the families had significant difficulties. However, delusional youths more often came from families with single mothers and absent fathers (three on four). The absence of intrafamilial violence in delusional adolescents contrasted with the situation of individuals who were judicialised for radical conduct. Likewise, we observed exacerbated family conflict or psychological control (found in most judicialised youths) for only 1 delusional individual. Judicialised adolescents had a much more conflictual family environment, and the members of this group had more manipulative relationships and psychological control.

#### Traumatic Experiences

By looking for traumatic experiences in delusional adolescents' life histories, we found two cases of psychological type I trauma (only one event). In addition, we observed three occurrences of type II trauma (repeated and lasting events) (Table 4).

For the convicted adolescents, we identified four cases of type I trauma. The following types of type II trauma were found in 14 of the 16 adolescents: intrafamilial violence, sexual abuse, continuous psychological pressure related to an affiliation with a sectarian group, exacerbated conflict and environmental instability caused by the parents' separation, the absence of a paternal figure because of imprisonment, and parents exhibiting psychiatric pathology. As we stated before, 5 of these 16 adolescents had suffered from intrafamilial violence, especially on their father's side, and extrafamilial sexual abuse in childhood (*N* = 3). In two other cases, we suspected abuse, but this experience was not reported.

In sum, the presence of type II trauma clearly predominated among adolescents in our sample, regardless of whether they had delusions.

### Psychopathological Analysis

In this part, we present the results from our psychopathological analysis based on a psychodynamic approach. We organized this analysis into three main themes: i) speech in relation to radical conduct, ideology and the group; ii) relational modalities, conflict management and defense mechanisms; and iii) perception of ideals and symbolic laws.

#### Speech in Relation to Radical Conduct, Ideology, and the Group

Here, we present the salient elements of adolescents' speech about their radical conduct and the contact they had made with others involved in radicalization. The different stories are not comparable, as they changed according to when they were collected. Indeed, adolescents' speech changed when their radical conduct appeared, when they were convicted, if imprisonment occurred, and in the case of individual and/or family educational and psychological support. Overall, as time passed between the subject's radical conduct and the interview, the subject's speech tended to become more disassociated from ideology and then, eventually, from group members.

For the delusional group, their relation to Islam and its ideology were very different from those of the convicted group. They did not mention or have any contact with members of a terrorist organization, but they all talked about fighting in the name of Islam in their own way. Subject 8, who had an East Asia non-Muslim cultural background, constantly spoke about the 9/11 attacks and expressed anti-Semitic statements. However, he expressed his claims with inappropriate affect, smiling in a friendly way, in a dissociative state. When he was alone, he spoke in soliloquies. When he was invited to talk about his conversion to Islam, he said, “*There are Muslims in the country where my family comes from, you know?... I don't eat pork because it isn't good for the stomach…* [stares at the care professional for a long time while he smiles inappropriately].” Subject 13 had a delusional syndrome that had emerged in relation to substance abuse. He expressed very persecutory and suspicious thoughts and behavior. As such, he threatened all the care professionals because they were “unbelievers,” telling them they would be punished by Allah and all his brothers in Islam. He also presented with megalomaniac tendencies and explained how he knew things that we ignored because he could speak and read Arabic, Hebrew and Aramaic. He also talked about the apocalypse. Subject 18, who had converted to Islam, experienced a psychiatric collapse while reading about the conflict in Syria and Islam on the internet. He went through an episode of mystical delusion and referred to redemption and his will to fight against “unbelievers” who threatened Muslims around the world. He talked about hidden information (as in a conspiracy) and God's path to protect him from temptation. Finally, he attempted suicide via defenestration and yelled “Allahu Akbar” before jumping. Subject 20 had a similar mystical delusion. His psychiatric collapse started after he had been the victim of an extremely violent racist attack on the street. All four subjects stopped their radical conduct as soon as their delusional symptoms were treated.

Regarding the 16 judicialised adolescents, we noticed that during the interviews and meetings, they showed a form of retrospective insight and constructed narratives about their radical conduct. They usually needed 1 year of hindsight; it took even longer for a few of the subjects. The adolescents we met less than a year after their radical conduct (*N* = 6) did not express this form of insight; they demonstrated either current adherence or a form of confused ambivalence, indicating that the personal conflict that brought them to a violent ideology was still relevant. For males, we identified three positions: 1) they still believed in the ideology; 2) they had distanced themselves from the violent ideology but remained in an omnipotent position; or 3) they explained that they had been “*tricked*” or “*fooled*” because they were young and inexperienced.

Subject 17, 6 months after radical conduct, stated, “*I was arrested because I was talking to radicalised people... radicalised for them! Not for me! For me, they were just Muslims. (…) I wanted to go to Syria to fight Bashar El-Assad because he was killing his people. In addition, to live with my religion. (…) ISIS people are slightly too barbaric... They consider all those who are not with them to be miscreants... I am more attracted by Al-Qaeda now*.

These adolescents often remained deeply attached to certain members of the group or to memories of this period, eventually missing their time with the group but also worrying about what the group members might think of them in the future. For example, would they be perceived as traitors?

Subject 14, a year-and-a-half after he had displayed radical conduct, made the following comment after a question was raised about an important person for him in the group*: “Yes, but I won't say his name, but he was a reference. I'm not in contact with him right now; since prison, nothing... I just don't want to create any problems for him. Maybe he changed, maybe not....”*

Subject 15, a year-and-a-half after he had engaged in radical conduct, made the following statement just after being asked about his relationship with the group: “*I was on Facebook; they were my friends and acquaintances, and we were playing a video game. One of them was a “brother,” he knew lots of things, and we agreed about many things, like being against suicide. (…) Then, there was a kind of euphoria, too many people from everywhere... I was the administrator of Facebook groups with 80,000 members; they were fun groups. In fact, many different opinions were expressed. (…) I was also able to guide another adolescent who had the same origins as me, and I was the one who convinced him not to go to Syria and to go to learn about the religion in Morocco instead.”*

These adolescents almost never mentioned their recruiters. On the other hand, they recalled moments of complicity or exaltation with their groups' peers with pleasure or nostalgia or moments with younger and inexperienced youths with whom they played a dominant or guiding role.

Two young girls were able to talk about the recruiter with whom they had built an affective relationship.

Subject 3, a year-and-a-half after she had engaged in radical conduct, said, “*The situation was very complicated with my family. I had the feeling that no one loved me... Then, I met him because he was a friend of my sister. He made me join the group and the discussions on social media; he always took my side in front of everyone. He used to [speak well of] me and call me his “little sister”; he protected me.”*

Subject 4, a year-and-a-half after she had engaged in radical conduct, stated, “*I suddenly found myself completely alone while I was suffering from bullying by the popular girls in my class... I didn't try to contact him; another mobile was given to me, and I started receiving his messages. His words…he was just someone who cared about me and who wanted the best for me; I had nothing else to lose.”*

The other two girls were able to adopt a position of omnipotence while avoiding speaking about their recruiter.

Subject 1, a year after engaging in radical conduct, stated, “*Honestly, they did everything I told them; I was very important. Before, I was nobody; then I became someone.”*

Concerning their motivations for prescribing to a violent ideology, most of them talked about altruistic reasons (in particular helping the Syrian population) or, more broadly, the desire to fight against injustice (starting with injustice supposedly committed by France). They also said they wanted to live harmoniously based on their religion in a Muslim country. Except for 1 adolescent who admitted being fascinated by violence and life after death (Subject 7), the other 15 judicialised adolescents did not mention what attracted them to a group that openly used violence and domination. Additionally, many said that they had tried to contact jihadists after seeing sensationalist reports about them on television.

These adolescents almost automatically remembered that before engaging in radical conduct, they had felt empty and lonely (an experience of abandonment) while facing the challenges of adolescence. Either through a review of their life experiences or through clinical examination, we noted dependency issues in their relationships, as if they were looking for an external reference point to hold onto.

#### Relational Modalities, Conflict Management, and Defense Mechanisms

In this subsection, we describe how the adolescents interacted with health professionals during the interviews, with the assumption that it provides indications about how they interacted and connected with others.

In the delusional group, the four youths had difficulty connecting with others. During their delusional syndrome, they were very suspicious and interpretative (except for Subject 8, who tried to create a reaction in his interviewer as a way of being present in the relationship), while the dissociation syndrome was apparent. When the delusional syndrome was treated with antipsychotics, the three subjects with schizophrenia felt more apathetic and tried to deal with their surrounding reality in a better way. Subject 13 had a better connection to reality but manifested relational modalities typical of attachment disorders (he had suffered from abandonment issues throughout his life).

In the convicted group, the females showed two types of stances with professionals: two of them looked for support from the practitioner, fearing abandonment, while for the other two, their fascination with the spectacular horrors committed by ISIS served as an urgent way to seek help. All female subjects were easy to communicate with. For 6 of the 12 males who had been prosecuted, the interactions were marked by a desire to fulfill the professionals' expectations of them, looking for a conformist adaptation. They talked about societal phenomena or politics and always seemed to be seeking approval by trying to look intelligent and to satisfy the adult. Three others manifested mistrust and hostility from the first interview, establishing an immediate power struggle dynamic. These three subjects had fewer similarities among them. Subject 9 expressed trivialization and avoidance (escapist behavior, several missed appointments). Subject 12 appeared to submit in an alarming way (a false sense of self), on the one hand showing extreme solicitude in the interview with the psychologist, but on the other hand, preparing to commit an extremely violent act. Subject 5 was compliant at the beginning; he quickly expressed himself in a grandiose way, while mythomania became apparent (untruthful claims, attempts to incite worries and fascination about him). Despite the diversity of these situations, the progress of many of these follow-ups indicated that youths could move from an initial mistrust to a request for orientation. Conversely, they could also go from a relationship based on seduction (or even fusion) to a feeling of persecution and massive rejection. Therefore, it is not relevant to categories the relational modalities of each subject. It seems more appropriate to consider a set of psychopathological constructs specific to these adolescents.

Beyond individual differences, these cases highlight a relational mode marked by the search for an exclusive relationship. This was shown by loyalty issues and unequivocally Manichean opinions. Relationships with professionals were contrasted: while some youths perceived professionals as models (showing a desire to become a doctor, a psychologist, an educator, or nurse) and listened to their words with a sense of idealization, others felt persecuted by them and saw the care situation as a form of humiliating submission. They all shared an incapacity to maintain the appropriate distance; they engaged in an exclusive relationship of submission or domination toward the other person. Thus, there was often an initial request for guidance followed by an attempt to escape from the relationship. As a consequence, conflict could not easily develop in the relationship.

From a psychodynamic perspective, the defense mechanisms mostly used by these 16 adolescents, such as trivialization and rationalization (*N* = 13), as well as inhibition and restriction (*N* = 6), were supposed to avoid conflict. We also noted several narcissistic defense mechanisms, such as the adoption of an omnipotent posture or a megalomaniac position (*N* = 6) or the switch between idealization and deidealization in the way the subjects dealt with their environment (*N* = 5). For example, a female patient would idealize her mother and strongly criticize her best friend; then, in the following session, she would criticize her mother and say that she and her best friend shared a common destiny. Similarly, many adolescents would idealize ISIS and then criticize the organization in favor of another Islamic practice or a new group, idealizing it in the same way. For half of these adolescents, we noticed a tendency to act out, independent of their radical conduct (*N* = 8). These actions consisted mainly of escapist tendencies but also clastic tantrums or heteroaggressive acts. Some of them employed hypomanic defenses characterized by excessive joviality, an inappropriate light tone, and use of humor (*N* = 4). For others, we found perverse defenses in their relationships, marked by a satisfaction to lie and manipulate (*N* = 4). Finally, we identified obsessive behaviors in three of these adolescents, who ultimately adopted an ultra-ritualized practice of their faith (*N* = 3).

#### Perception of Ideals and Symbolic Laws

Idealization seemed to play an important role for these adolescents. Additionally, their integration of symbolic law appeared impaired.

During their delusional syndrome, the members of the delusional group expressed a fascination with a powerful representation of God, as well as a feeling of accessing hidden and powerful secrets in a mystical way. They all had difficulty understanding societal and symbolic laws and limitations. However, this challenge was clearly linked with their psychiatric disorder, especially their faulty conception of reality.

Regarding idealistic expectations in the convicted group, we observed repeated exaltation due to a feeling of belonging in a group where they were valued, where they did not feel patronized, and where great promises were made to them. One adolescent talked about becoming an emir in ISIS as becoming “*a leader who commands warriors fighting for him*.” Many of the subjects mentioned the grandiosity of the ideal community they had joined *(“A place where everyone is accepted, no matter where they come from!”*), while others talked about intolerable images of abuse or war they had witnessed. Many also seemed driven by an attachment to an identity of humiliation and oppression from which the only dignified perspectives remaining were engaging in rebellion and fighting against injustice. Most of the subjects mentioned that adhering to the ideology and joining the group members were totally fulfilling and allowed them to fill the emptiness they had felt since adolescence.

Moreover, a third of them no longer were concerned with sharing ideals or fighting against injustice but were more fascinated by the acts of violence committed by ISIS and showed a form of attraction to places or groups where this domination was exercised. In fact, even if these adolescents struggled to talk about the ideology and the group, we noticed a form of fascination and attraction to omnipotent and grandiose figures. Sometimes, they added a dimension of rebellion and armed struggle against injustice (*N* = 6) or referred to secret knowledge, as in conspiracy theories (*N* = 2). For others, a fascination with domination appeared (*N* = 7), including violence (*N* = 4). For those fascinated by violence, three of the four adolescents had been sexually abused in childhood (we suspected such abuse for the fourth adolescent).

Regarding how the adolescents related to symbolic law, in their speeches, we found recurring themes such as the question of transgressions (committed and/or suffered), as well as the need to rebel against injustice. However, none of the adolescents had completely integrated what was not allowed but instead were constantly trying to justify themselves. A female patient said during a session, in a very serious way, that she had done worse than ISIS decapitations and mass killings by running away from home. Another young girl with abandonment issues explained several times that there are no valid rules, and the only important thing is the law of the strongest (the challenge for her was being on the “right side,” meaning the strongest side). Several times, we noted a lack of empathy in some of the youths toward their relatives, and paradoxically, we sensed excessive empathy toward some individuals of the jihadist group or toward Muslims in Syria.

During their care, we carried out projective tests with six subjects using the Rorschach test and Thematic Apperception Test (51, 52). There were many results, so we highlight some details to illustrate how adolescents related to symbolic law. For example, on the Thematic Apperception Test, individuals must elaborate a story based on images representing conflict and/or affective situations between members of different genders and ages. None of the adolescents mentioned a third party—or a figure representing the law or limits—as is normally expected. In their answers, where avoidance and rationalization predominated, the only relationships mentioned were dual, showing themes of domination or fusional dynamics within an early mother-child relationship. In two cases, we witnessed a perverse form of enjoyment when the transgression of the law was mentioned.

Finally, none of the 16 adolescents expressed a feeling of guilt during the follow-up, either toward their family, their environment, or the members of the group. This seemed worrisome, especially knowing that such feelings have appeared on several occasions in the context of the follow-up of adolescents who did not manifest actual radical conduct (the most common situation being an adolescent converting to Islam whose family was not Muslim; see Figure 1).

In sum, we believe these adolescents were attracted by a grandiose ideal they perceived as accessible through attachment to an individual and/or group ideology. We also believe they did not efficiently integrate the symbolic law and the prohibitions that come with it.

## Discussion

### How These Results Match the Trajectories Highlighted in the Literature

In this section, we aim to verify whether our results correspond to hypotheses about specific trajectories present in the literature depending on the presence of a delusional syndrome, the gender of subjects and the existence of a specific profile of adolescents more attracted by violence. The small size of our sample does not allow us to generalize these hypothetical trajectories. Our aim is to verify whether these specificities appear as a clear distinction within a small sample and whether they are operational in a clinical setting.

#### Possible Trajectories Associated With Delusional Syndrome

In terms of individual trajectories, the main difference we can highlight in these results is between the delusional group and the convicted group. Even though the delusional group was small, we found several specificities in their radical conduct (acute and disappearing quickly after psychiatric treatment). None of them had been in contact with a member of a terrorist organization. They often had converted to Islam and lived in single-mother families with an absent father. Additionally, we did not find any intrafamilial violence or sexual abuse in their personal histories, which was in contrast to the convicted group. Their family environment was less conflict ridden and less characterized by psychological control. From a clinical perspective, these four youths corresponded to the population that usually receives psychiatric adolescent therapy for psychotic collapse. In comparison with the convicted group, their radical speeches were incoherent, disordered and illogical. It appeared that their radical conduct originated in a delusional syndrome that developed in association with a societal theme or a collective concern, as often occurs in delusional syndromes. Therefore, a clear distinction appeared between adolescents with a delusional syndrome and those without it in terms of the way that radical conduct appeared and disappeared.

At this point, it is interesting to refer to Meloy's distinction between “delusion,” “obsession” and “extremely overvalued belief,” three types of cognitive-affective drivers of pathological fixation preceding most cases of targeted violence (57). The four subjects of the delusional group evidently possessed the characteristics of “delusion,” defined as a “*[nonfactual] certainty which is fixed, false and idiosyncratic*” and a “*belief that is not ordinarily accepted by other members of the person's culture or subculture*” (57). Even if these four subjects based their pathological fixation on jihadist content on the internet (which can be considered the beliefs of a subcultural group), their expressions clearly corresponded to a delusional syndrome. Furthermore, most of these four adolescents referred to idiosyncratic content that could not be shared by a jihadist group (e.g., Subject 18 had yelled “Allahu Akbar” before attempting suicide, which is clearly forbidden in Islam; Subject 13 threatened all “unbelievers” but at the same time talked about secret knowledge he had because he could supposedly read Hebrew and Aramaic, which was obviously false and had no consistency with any Islamic ideology that we know of). In addition, these adolescents had no contact with members of a terrorist organization and, as far as we know, had simply watched videos without truly interacting at length with anybody who shared at least some of their beliefs.

On the other hand, the other 16 “convicted youths” radical conduct corresponded to “extremely overvalued beliefs,” defined by Rahman as beliefs “*shared by others in a person's cultural religious or subcultural group. The belief is often relished, amplified, and defended by the possessor of the belief and should be differentiated from an obsession or delusion. The belief grows more dominant over time, more refined, and more resistant to challenges. The individual has an intense emotional commitment to [his or her beliefs] and may carry out violent behaviour in [their] service*” (58). These distinctions are also critical regarding treatment recommendations. The authors explained that youths with delusions “*are amenable to treatment with antipsychotic medications, and most such patients will benefit from antipsychotic drugs*” (57). To treat extremely overvalued beliefs, the authors asserted that antipsychotic drugs do not appear to alter these beliefs, and they recommended cognitive-behavioral therapy, as well as family and group therapy. They also stated that classical “clinical checklists” for psychiatric disorders will often fail to identify individuals who are demoralized, grief-stricken, or angry. The “*examiner should begin by taking a comprehensive history in chronological fashion to arrive at a conclusion based on temperament, cognitive capacity, and motivation*” (57). To a certain extent, these recommendations meet the psychotherapeutic recommendations we present below.

The proportion of individuals with mental illness in our sample (20%) is not representative of the population of adolescents associated with radicalization in France. Our sample is too small, and there is bias because our study is based in a single child psychiatry department. However, even with these important limitations, it is interesting to note that this proportion remains low. We also observed that, compared adults participating in previous studies (6, 7), the four youths with mental illnesses in our study seemed barely linked to members of violent extremist groups. Even if this group factor is difficult to objectify among adolescents in psychiatry, it is important to keep in mind that the majority of psychotic disorders appear in young adults (59). Indeed, Subjects 18 and 20, who belonged to the delusional group, were already adults (18 and 20 years old) when they were engaging in radical conduct. Moreover, violent extremist groups tend to recruit adults because they seem to be more useful, reliable, and suitable for clandestine activity (17, 18), even if the internet has helped them to spread violent ideologies in a broader way. It is also important to note that it is not possible to generalize the trajectories of all individuals who have a delusional syndrome and manifested radical conduct based on the four subjects we examined in this study. For instance, we know that a few cases of individuals with this clinical picture can, after the treatment of their delusional syndrome, still demonstrate extreme overvalued beliefs about radical ideologies. The mediatized case of Nathan Chiasson is a good illustration (60). This 22-year-old man who committed an attack with a knife in the name of Islam had a psychiatric history since adolescence. It appears that after he was treated for schizophrenia, he still endorsed a radical ideology and that he had a girl friend who also shared this ideology. It appears that we did not encounter this kind of trajectory in our sample. Further investigation is needed to better understand the links between psychiatric disorders and radicalization among adolescents.

#### Possible Trajectories for Adolescent Girls

Two qualitative studies previously showed gender differences among radicalised youths. For example, both studies highlighted the “sleeping beauty” profile in some girls engaged in radicalization: a female in search of an ideal husband/love (20, 25). The analysis of the group of 16 convicted adolescents underlined several differences between females (*N* = 4) and males (*N* = 12). There was a difference concerning ideology appropriation, which entails clearly knowing that one's sexual identity is decisive during adolescence and that ISIS clearly differentiates roles assigned to each gender. We noted four major differences: 1) more intellectualization among boys, 2) the fantasy of impure sexual desire among girls, 3) more frequent research on ascetic behaviour among girls, and 4) more mentions of romantic aspects of their relationships with group members among girls. However, this latter characteristic (romantic aspects) was only obvious for two of the four girls. The two other girls were much more vindictive and displayed a fascination with ISIS exactions. Therefore, this “sleeping beauty” profile seems to appear only among some of the adolescent girls who embrace the ISIS ideology in France. Furthermore, the follow-up of these two girls, motivated by romantic aspects in our sample, indicated that they responded very well to educational and psychological support, as suggested by qualitative studies (20, 25). Even if these results correspond to the existence of a specific “girl profile” called the “sleeping beauty” profile in a previous study, it does not mean that every adolescent girl with radical conduct corresponds to this profile, as half of the girls of our sample did not match this profile. Further investigation is needed to better understand the correlation of gender and radicalization trajectories.

#### Possible Trajectories of Adolescents Attracted by Violence

One of the studies we mentioned earlier (27) underscored a trajectory with worse outcomes (still being radicalised or having reached the Islamic State). Individuals on this trajectory are more often part of a local context. They are especially vulnerable to external control in the form of relational clinging (22). Motivational aspects linked to *interest in violence and weapons, adventure, fighting, “male values,” lack of self-esteem*, and *a lack of interest in searching for tenderness* correlated significantly with a worse outcome (25). Our results are consistent with the description of these possible trajectories, as we detected more alarming profiles in the sample of youths whose lives had been marked by violence (against them or initiated by them). If we examine the six subjects who had had physical encounters with members of the violent extremist group, we found that—except for the girls (Subjects 1 and 3)—they all had a worse follow-up status. Two boys expressed a clear fascination with violence and domination (Subjects 6 and 12); three were still radicalised after a year of educational, judicial and psychological support (Subjects 6, 12 and 17); and one had reached Syria with his older brother (Subject 11), which corresponds to the characteristics of the worse follow-up status mentioned above. Finally, our results imply that these subjects—who were more fascinated by violence and relational domination—had been exposed to sexual abuse or violence during childhood, as shown previously. These youths were also more difficult to provide care for. These results seem to match the existence of a more worrisome profile of adolescents who are more attracted by violence. Additionally, our results seem to indicate that adverse childhood experiences, especially sexual abuse, are related to this profile. Further investigation focused on adverse childhood experiences in a larger sample of individuals attracted by violence and resistant to deradicalisation programmes is needed to better understand the specifics of this radicalization trajectory.

### The Contributions of a Psychopathological Approach

In this section, we summarize our chief clinical observations and develop the central hypothesis we formulated regarding the influence of the adolescent period and relationships marked by psychological control. These considerations are based on our professional care and experience, and even if they cannot be taken as empirical statements, we believe that they could provide input for the international debate about adolescents' radicalization and offer indications for professionals who deal with this phenomenon. For this reason, we conclude this part with care recommendations.

#### Adolescence and Radical Conduct

The insights of this case series underline the difficulties for youths who have upsetting experiences during adolescence. We noticed the recurrence of feelings of loneliness and school bullying before the appearance of radical conduct. The adolescents suffered from divided family functioning with exacerbated conflict, loyalty conflict, narcissistic wounds, feelings of injustice, and psychological control. These youths also played out relational modalities with the professionals around them (total rejection or relational control). Thus, it seemed difficult for them to find a way through the separation and individuation issues of adolescence (61, 62). This explains why they could not accept a symbolic law that establishes limits but that supports them and provides them with reassuring bearings. Their relational style during consultations revealed their difficulties in appropriate and internalized experiences that they went through in their usual environments. Furthermore, their relational attachment indicated a major external dependence and difficulty finding a suitable distance in social relations. Therefore, issues facing youths seem to be an important factor in their identification with a violent extremist ideology and group, as clinical literature has noted (28–30).

#### An Ideological and Group Proposition

In addition to the challenges facing the adolescents involved in the present study, violent ideology and radical conduct played a role in their psychological functioning, allowing them to adopt a spectacular/grandiose identity. The use of intellectualization and the omnipotence of idealized thinking enabled them to deny the upheaval of their changing bodies during puberty. Radical groups ask youths for commitment and submission and, in exchange, offer them a place in the group and a sense of belonging (4, 63). They seduce and flatter teenagers, and they talk about fighting, being warriors, getting married, and becoming parents. The tyranny of the ideological dogma guarantees the control of motivation with standardized group behaviors but also allows—and even encourages—the expression of aggression and violence. In this way, the ideological offering and the way these youths integrate it gives them a temporary solution to their crises; it allows them to act out conflicts that they cannot manage in their internal psychological worlds.

#### Clinical Hypothesis About Radicalization Among Adolescents

These observations allowed us to formulate a hypothesis about convicted adolescents: Radical and violent ideologies release these adolescents from family psychological control and lead them to a connection that remains familiar, as previous clinical comments have suggested (31, 32). Many of the convicted adolescents in our sample seemed to experience early psychological control in their families (43), whose members also seemed to present a form of dependence and for which the “third-party function” of the law was fragile. In these families, the youths seemed to be alienated by notions that they used to fight against their disappointed ideals. Dependence, psychological control, and relational attachment appeared to be the main characteristics of this functioning. We believe that these adolescents cannot give up the ideals and grandiose expectations of early childhood. We perceive that they tried to control their massive aggressive drives, even if doing so implied alienating themselves. They jumped from one guru to another, trying to escape control but, paradoxically, also seeking a mentor who could fill their expectations and soothe them. We wonder if these successive submissions and detachments would favor, step by step, a path toward psychological independence or if the youths would ultimately become attached to a tyrant. In this respect, their young age allows us to remain hopeful for a positive outcome for most of them, assuming their environment will detect this potential and help them take advantage of it.

#### Care Recommendations

For patients whose radical conduct appeared during a delusional syndrome, the classical support offered in child psychiatry seems to be enough to make this radical conduct disappear in most cases. However, some more limited delusional subjects show an extreme overvalued belief after psychiatric treatment while they have contacts with individuals who share their radical ideology. Therefore, practitioners should be aware of these particular subjects who may commit violent acts even if their psychiatric pathology is stabilized.

For other adolescents, psychotherapeutic support, in addition to educational and judicial follow-up, seems appropriate for both the youth and the parents. As previously stated, this is consistent with Meloy's recommendations regarding extremely overvalued beliefs (57). The main purpose of this support is to favor the emancipation attempts that these adolescents demonstrate. The objective is to support them throughout adolescence, to help them find a sense of belonging in their environments and to regulate family transactions in more harmonious ways so that the external struggle they display can be internalized. During our psychotherapeutic follow-up, we tried to help adolescents build their identities and to present themselves to others in a more independent, measured way by giving up a position of omnipotence and domination (32, 41).

Such an approach requires that the therapist adopt a third-party stance aside from the family and the judicial and security institutions. It is important to maintain this position to avoid the temptation of Manichean and binary thinking, which is often frequent in these situations. The follow-up of these adolescents often generates power relationships, disapproval, virulent attacks against the therapist, and/or, conversely, a form of relational attachment in the pursuit of fusion or submission to the therapist. It is essential for the therapist to be vigilant and aware of these extreme positions and not to fall into two main pitfalls: the satisfaction of the individual who inculcates knowledge or the temptation of being in a judicial or security position. It is necessary for therapists to keep in mind during consultations that we are also subject to real and symbolic laws and that we have no control over the patient's opinions or judicial decisions that are made.

The experience of psychotherapeutic follow-up underscores the importance of starting from political, religious, and social issues that these adolescents bring to consultations. Gradually starting from these themes, the patient can develop more intimate issues. The therapist can invite the patient to adopt a point of view that the patient can use that is neither that of an enemy nor that of a guru but that of a third party. For adolescents to be able to take advantage of this proposal, it is necessary for follow-up to continue over time. Our experience has shown the need for at least 1 year of educational and psychotherapeutic support to allow these adolescents to reflect on themselves and on their history, at which point they can eventually utilize the support offered to them.

Furthermore, even though we did not collect our results as part of an educational follow-up, we insist on the essential role of regular educational support for these youths. Indeed, we found that adolescents whose progress was more optimistic were often those for whom stable educational support was provided over the long term (26).

Finally, we recommend that parents and teachers who may be worried about a “radicalization process” among adolescents first try to understand the function of their conduct. What is the adolescent trying to warn us about himself or herself? It seems important to keep in mind that, most of all, adolescents are dealing with themselves and their environment. They are in a period of substantial changes, and it is important to consider that this dynamism and liability of adolescence remain the best opportunity for a favorable outcome (27). If adults surrounding an adolescent have serious “radicalization” concerns, they should be transparent about it with the youth and keep trying to understand and maintain the relationship as much as possible with him or her. Additionally, they should ask for help regarding these concerns. For security concerns, adults should contact security services that address radicalization, and for their misunderstanding of this conduct, they should seek help from specific programmes such as ours and/or specialized associations. In some of these situations, adults will find support with a religious guidance, while in others, they will find help through therapeutic work.

## Limitations

This study has several limitations, such as the use of a small sample and a qualitative and retrospective methodology. However, the marginal aspect of radicalization and the lack of publications offering a psychopathological perspective on radical conduct among adolescents justify this choice. It would be interesting to extend the present research through other studies that create a dialogue between public health studies (64–67), investigations using standardized scales in medium-sized samples of adolescents who are “at risk of radicalization” or who have become “radicalised” (26, 68), and studies with a psychopathological perspective based on concrete case reports (28, 29, 32, 33).

Another important limitation is that all radical conduct was rooted in ideologies that were claimed to belong to Islam. Other radical conduct (e.g., extreme right or extreme left) would have been interesting to explore, but we have not encountered such situations, certainly because of the current geopolitical and societal climate (69, 70). Moreover, violent ideologies of the extreme right concerning national identity and racism do not motivate the same concerns within Western societies (71, 72). It is critical to investigate different types of violent ideologies because security agencies, the media, and public opinion are more likely to perceive Islamist-inspired extremism as terrorism and right-wing extremism as a psychopathological problem (73).

Finally, it is crucial to remember that we explored adolescent radical conduct in a situation where youths were opposed to their families' beliefs and ideologies. When violent ideology is transmitted by the parents and the family, radical conduct falls under different psychopathological considerations. It would also be interesting to compare these two types of radicalization in future research.

## Conclusion

The examination of detailed clinical characteristics of adolescents who have manifested radical conduct tends to confirm the relevance of differentiating individuals with delusional syndrome from those with an “extremely overvalued belief” (58) who are influenced by members of a terrorist group. This significant distinction also appears necessary because, as mentioned in the literature (57), the suggested treatment differs for each situation, as we verified with our experience (radical conduct during delusional syndrome appears to be well treated with antipsychotic drugs). Our study also establishes interest in certain profiles and factors described in the literature (20, 22, 25, 27, 74, 75). The “sleeping beauty” profile (romantic motivation) resonated with two adolescent girls in our sample; their educational and psychopathological follow-up led to very good and rapid results, which is in line with large outcome studies (25, 27). The importance of proximal/neighborhood phenomena also appeared in some of the individuals we examined as a factor for worse follow-up status since they had more difficulty giving up their identification with the extremist group/ideology (27). Furthermore, the individuals in our sample who were more attracted to domination and violence were more difficult to treat, as suggested by the analysis of motivational aspects in a previous study (25). Our results also imply that this more worrisome profile of radicalised adolescents concerns individuals who are more exposed to sexual abuse and violence in childhood. Future investigations based on a larger sample should help confirm whether these adverse childhood experiences, proximal exposure to radicalization, and fascination with violence and domination are negative prognostic factors for adolescents in deradicalisation programmes. Finally, our psychodynamic analysis permitted us to formulate hypotheses about nondelusional radicalised adolescents. The central hypothesis is that their relational modalities are characterized by psychological control and domination, leading to difficulties facing youths and situations where violent extremist propositions soothe adolescents, as Kruglanski's significance theory suggests (4).

## Data Availability Statement

The original contributions presented in the study are included in the article/supplementary files, further inquiries can be directed to the corresponding author.

## Ethics Statement

The studies involving human participants were reviewed and approved by the Ethics Evaluation Committee of the INSERM (Institut National de la Santé et de la Recherche Médicale) Institutional Review Board (IRB00003888, IORG0003254, FWA00005831). Written informed consent from the participants' legal guardian/next of kin was not required to participate in this study in accordance with the national legislation and the institutional requirements.

## Author Contributions

NC and EL contributed to conception and design of the study. NC organized and explored the clinical data and wrote the first draft of the manuscript. DC and AO checked each analysis grid to validate sociodemographic and clinical data. NC, EL, and CT made the qualitative analysis. EL and DC wrote sections of the manuscript. AO, CT, and DC helped with translation to English. All authors contributed to manuscript revision, read, and approved the submitted version.

## Funding

This study was supported Laboratoire PCPP- EA 4056, Institut de Psychologie Université de Paris C.A.R.P.I.J. CENTRE D'ACTIVITES ET DE RECHERCHES EN PSYCHIATRIE INFANTO-JUVENILE.

## Conflict of Interest

The authors declare that the research was conducted in the absence of any commercial or financial relationships that could be construed as a potential conflict of interest.

## Publisher's Note

All claims expressed in this article are solely those of the authors and do not necessarily represent those of their affiliated organizations, or those of the publisher, the editors and the reviewers. Any product that may be evaluated in this article, or claim that may be made by its manufacturer, is not guaranteed or endorsed by the publisher.
